# Multidisciplinary Investigations of Sustained Malaria Transmission in the Greater Mekong Subregion

**DOI:** 10.4269/ajtmh.21-1267

**Published:** 2022-10-13

**Authors:** Liwang Cui, Jetsumon Sattabongkot, Pyae Linn Aung, Awtum Brashear, Yaming Cao, Jaranit Kaewkungwal, Amnat Khamsiriwatchara, Myat Phone Kyaw, Saranath Lawpoolsri, Lynette Menezes, Jun Miao, Wang Nguitragool, Daniel Parker, Suparat Phuanukoonnon, Wanlapa Roobsoong, Faiza Siddiqui, Myat Thu Soe, Patchara Sriwichai, Zhaoqing Yang, Yan Zhao, Daibin Zhong

**Affiliations:** ^1^ Department of Internal Medicine, Morsani College of Medicine, University of South Florida, Tampa, Florida;; ^2^ Mahidol Vivax Research Unit, Mahidol University, Bangkok, Thailand;; ^3^ Myanmar Health Network Organization, Yangon, Myanmar;; ^4^ Department of Immunology, China Medical University, Shenyang, China;; ^5^ Department of Tropical Hygiene, Mahidol University, Bangkok, Thailand;; ^6^ Department of Epidemiology, University of California at Irvine, Irvine, California;; ^7^ Department of Medical Entomology, Faculty of Tropical Medicine, Mahidol University, Bangkok, Thailand;; ^8^ Department of Pathogen Biology and Immunology, Kunming Medical University, Kunming, China;; ^9^ Program in Public Health, University of California at Irvine, Irvine, California

## Abstract

In the course of malaria elimination in the Greater Mekong Subregion (GMS), malaria epidemiology has experienced drastic spatiotemporal changes with residual transmission concentrated along international borders and the rising predominance of *Plasmodium vivax*. The emergence of *Plasmodium falciparum* parasites resistant to artemisinin and partner drugs renders artemisinin-based combination therapies less effective while the potential spread of multidrug-resistant parasites elicits concern. Vector behavioral changes and insecticide resistance have reduced the effectiveness of core vector control measures. In recognition of these problems, the Southeast Asian International Center of Excellence for Malaria Research (ICEMR) has been conducting multidisciplinary research to determine how human migration, antimalarial drug resistance, vector behavior, and insecticide resistance sustain malaria transmission at international borders. These efforts allow us to comprehensively understand the ecology of border malaria transmission and develop population genomics tools to identify and track parasite introduction. In addition to employing in vivo, in vitro, and molecular approaches to monitor the emergence and spread of drug-resistant parasites, we also use genomic and genetic methods to reveal novel mechanisms of antimalarial drug resistance of parasites. We also use omics and population genetics approaches to study insecticide resistance in malaria vectors and identify changes in mosquito community structure, vectorial potential, and seasonal dynamics. Collectively, the scientific findings from the ICEMR research activities offer a systematic view of the factors sustaining residual malaria transmission and identify potential solutions to these problems to accelerate malaria elimination in the GMS.

## INTRODUCTION

Malaria is a mosquito-transmitted parasitic disease that occurs primarily in impoverished tropical and subtropical areas of the world. In the Greater Mekong Subregion (GMS), which consists of Cambodia, China’s Yunnan and Guangxi provinces, the Lao People’s Democratic Republic (Laos), Myanmar, Thailand, and Vietnam, malaria has been one of the most severe public health issues, hampering socioeconomic development.^1^^–^^3^ Recent decades have welcomed bourgeoning economic growth and significant improvement in public health in GMS countries. Driven by increasing political commitment and motivated by recent achievements in malaria control,^3^^,^^4^ the six GMS nations have endorsed a regional malaria elimination plan with an ultimate goal of eliminating *Plasmodium falciparum* malaria by 2025 and all malaria by 2030 in all countries of the GMS.^5^ Recently, after 3 years with no indigenous malaria cases, China was certified as malaria-free by WHO, marking a major success in the decades-long fight against this disease. However, various setbacks have been encountered in other GMS countries due to existing and emerging challenges (detailed in the following).

Malaria control and elimination rely on accurate and timely knowledge of the distribution of malaria incidence and prevalence, delivery of effective chemotherapy, and implementation of operative vector-management strategies. The complex and fast-evolving malaria epidemiology in the GMS is reflected in its immense spatial heterogeneity and the emerging dominance of *Plasmodium vivax*, a parasite species with remarkable resilience to conventional malaria control methods.^6^ In addition, artemisinin (ART) resistance in *P. falciparum*, detected initially in Western Cambodia a decade ago, has received augmented local and international concerns.^7^^–^^10^ Failure to contain ART-resistant parasites and the emergence of resistance elsewhere in the GMS escalated the urgency for a regional plan of malaria elimination.^11^^,^^12^ Further, the effectiveness of two core vector control interventions (insecticide-treated nets and indoor residue spraying) has been declining due to the development of insecticide resistance and increased outdoor biting of vectors.^13^^,^^14^ To address these problems, the Southeast Asia International Center of Excellence for Malaria Research (ICEMR) has developed a multidisciplinary program, aiming to understand how human migration, antimalarial drug resistance, and vector adaptations contribute to continuous malaria transmission at international borders so that integrative control strategies can be developed. To realize this scientific goal, we have strategically selected representative sentinel sites along the international borders of China, Myanmar, and Thailand, where malaria epidemiology is drastically different from each other. Using systems approaches and innovative technologies, we want to dissect the tripartite interactions among migrant human populations, diverse mosquito vectors, and multidrug-resistant (MDR) parasites to develop novel control strategies to propel the course of regional malaria elimination.

## EPIDEMIOLOGY OF BORDER MALARIA

### Spatial epidemiology.

**Figure 1.**
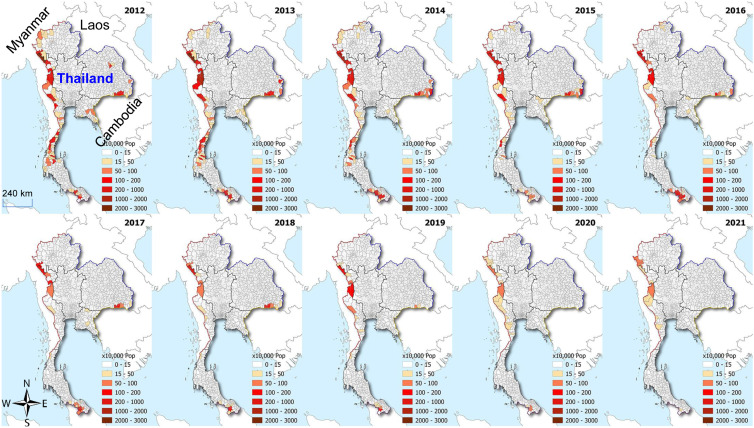
Spatial distribution of malaria incidence in the border areas of Thailand, 2017–2021. The figure illustrates persistent border malaria despite the gradual reduction of annual malaria incidence. Neighboring countries and scale bar are marked in one panel. This figure appears in color at www.ajtmh.org.

The distribution of malaria in the GMS exhibits extreme heterogeneity at both macro and microgeographical scales.^15^^,^^16^ The six GMS countries have advanced to different stages of malaria elimination, with Myanmar having the highest malaria incidence (almost 70% of the regional burden). Although border malaria (concentrated malaria transmission along international borders) is a shared phenomenon of each country,^17^ intensified control efforts have led to isolated pockets of malaria transmission.^18^ In Thailand, malaria has declined over the last several decades, but pockets of malaria transmission persist along the Thai–Myanmar border (Figure 1). Of the 927 border districts, 637 (69%) reported malaria incidence in the past 3 years and 307 (33%) in 2021. Similarly, during the final phase of malaria elimination in China, malaria in the border counties of Yunnan province displayed large spatiotemporal changes with incidence clustered in several hotspot townships.^16^ While the *P. falciparum* clusters shifted locations and cluster size each year, high-incidence vivax malaria clusters persisted.^16^ Within villages, malaria also exhibited evident transmission hotspots, probably depending on the local ecology of vectors.^19^^,^^20^

**Figure 2.**
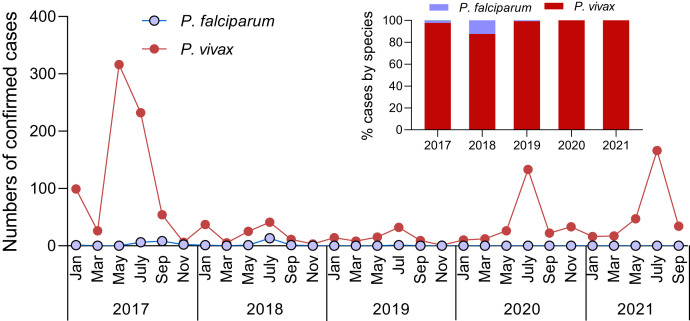
Dynamics of confirmed *P. vivax* and *P. falciparum* cases from passive case detection at the Laiza township hospital in Myanmar, 2017–2021. The Inset panel shows the dominance of *P. vivax*. The vivax malaria outbreak in 2016–2017 was effectively suppressed by vector-based control efforts (IRS and street fumigation). Case rebounds were noticed in 2020–2021, which may be due to reduced control efforts during COVID-19. This figure appears in color at www.ajtmh.org.

Another conspicuous change in malaria epidemiology is the increasing dominance of *P. vivax* malaria.^21^^,^^22^ Surveillance of clinical malaria cases at the China–Myanmar border detected an increase in the proportion of vivax malaria from ∼60% in 2011 to > 97% in 2016, with occasional vivax malaria outbreaks.^21^ Such a trend has persisted in more recent years (Figure 2). The proportional increase of vivax malaria is partially attributed to its ability to relapse, which requires 14-day primaquine (PQ) radical cure, a regimen with ubiquitously poor compliance. In a cohort of 7,000 village residents on the Western Thai border, we detected 410 malaria cases by microscopy in 6.5 years. Among them, 67 people had multiple malaria episodes within 1 year of the initial infection, and 60% of these recurring infections were due to *P.vivax*.^23^ The resilience of vivax malaria to conventional malaria control measures necessitates new tools for its elimination.

### Risk factors for malaria transmission.

Risk factors for malaria infection vary by parasite species, geography, and demographic attributes. *Plasmodium falciparum* is more geographically restricted and clusters in rural, remote areas with poor healthcare access—especially along borders. In much of the GMS, *P. falciparum* infection clusters in adult males who are exposed to the parasite through travel to hotspots of the disease (e.g., forested areas).^21^^,^^24^^,^^25^ Certain occupations (e.g., farming, military) bear a significantly higher risk of malaria while students have an increased risk of vivax malaria.^21^^,^^24^ Individuals with poor access to health services, with linguistic barriers, of ethnic minorities, and without citizenship may also have a higher likelihood of infection.^20^^,^^21^^,^^26^ For both vivax and falciparum infections, individuals who have a history of malaria infection are more likely to have subsequent infections.^21^^,^^24^ Housing characteristics are also related to the risk of infection, presumably associated with *Anopheles* permeability (open structures, building materials, distance to mosquito breeding habitats, etc.).^20^^,^^27^^,^^28^ In addition, housing can be a proxy for other factors like socioeconomic status, which influences occupation and access to healthcare. Identifying high-risk populations facilitates the implementation of targeted malaria control measures. Delivering health education messages to hotspot villages^29^ and malaria prevention packages to forest-goers and farmers staying in farm huts will help change risky behaviors and reduce malaria infection.^30^^,^^31^

### Malaria parasite detection and surveillance.

In low-endemic malaria settings in border communities, most *Plasmodium* infections appear to be asymptomatic and submicroscopic,^32^ requiring sensitive molecular tools for detection. We have demonstrated that submicroscopic infections can infect mosquitoes,^33^ constituting a critical reservoir for persistent transmission. In clinical settings, malaria diagnosis is routinely performed using light microscopy and rapid diagnostic tests (RDTs). RDTs have recently gained considerable traction in the GMS and play an indispensable role in evidence-based treatment, especially in hard-to-reach remote communities along international borders, where quality microscopy is often inaccessible. As most of the RDTs deployed in the GMS for *P. falciparum* are based on the detection of histidine-rich protein (HRP) 2 protein, our recent findings on the emergence of parasites with *pfhrp2* deletion in the Western GMS suggest potential challenges for the continued use of such RDTs.^34^^,^^35^ Consistent with the suboptimal performance of RDTs against nonfalciparum and nonvivax human parasite species found in Southeast Asia,^36^ we also demonstrated the failure of a conventional RDT to diagnose high-density (> 500 parasites/mL) acute febrile infections of *Plasmodium malariae* and *Plasmodium ovale* in the China–Myanmar border area.^37^

Microscopy and RDTs have limited utility for active surveillance because parasite densities in asymptomatic infections are often below their detection thresholds. Recognizing these limitations, we have conducted studies to compare potential new solutions, aiming to identify pragmatic tools for disease surveillance in the GMS. The recent advent of an ultrasensitive RDT (uRDT) for *P. falciparum*, having a detection limit 10 times lower than conventional RDTs, prompted the team to investigate its utility for active surveillance.^38^ Our study conducted in endemic areas of Myanmar demonstrated that uRDTs have approximately 20% increased sensitivity in detecting subclinical *P. falciparum* infections when compared with standard RDTs.^39^ Ultrasensitive RDTs still have lower sensitivity than molecular assays and are unlikely to identify all subclinical infections, but they are a promising improvement in our ability to monitor *P. falciparum.* The increasing predominance of *P. vivax* demands the development of uRDTs for this species.

The program evaluated several molecular diagnostics, including qPCR, nested PCR to detect parasite rRNA genes, nested reverse-transcriptase PCR (nRT-PCR) to detect parasite rRNAs, and capture and ligation-probe PCR (CLIP-PCR) to detect parasite rRNAs in cross-sectional surveys.^40^^,^^41^ The rRNA-based method has the highest sensitivity and rivals that of high-volume PCR,^42^ but the RNA detection requires a much smaller blood volume and is more suitable for active surveillance in many places. Applying nRT-PCR to finger-prick blood samples from community surveys in Northeastern Myanmar uncovered an infection prevalence of nearly 20% compared with 1% by light microscopy,^27^^,^^40^ further demonstrating the feasibility and the gain of using a sensitive molecular tool. As costs are one major impediment to molecular testing, a simple and flexible method of sample pooling was devised,^39^ which can be tailored to different endemicities. As most infections in areas approaching elimination are asymptomatic and submicroscopic,^33^ molecular surveillance in sentinel sites is essential for guiding targeted control practices, determining the effects of control measures, and monitoring the progress toward elimination. Further fine-tuning these molecular tools to differentiate the drug resistant and sensitive parasites in a clinical setting would also be crucial for timely adjusting drug policies.

### Migration and malaria introduction.

Border malaria poses a vital threat to malaria elimination and requires multinational cooperation.^43^ Heavy population flow along the extremely porous borders makes neighboring countries vulnerable to malaria introduction and reintroduction.^44^^,^^45^ Human migration may be partially responsible for the cross-national spread of ART-resistant strains with specific multidrug resistance genotypes.^46^ The association of a higher risk of malaria with the migrant population and those with travel to Myanmar highlights the significance of malaria introduction by migratory populations in the border region.^20^^,^^47^^,^^48^ Although passive case detection activity in the Southwestern border of China only showed strong evidence of imported *P. falciparum* malaria,^47^ subsequent genetic studies at the China–Myanmar border using microsatellite markers revealed genetically homogenous populations for both parasite species on both sides, indicating extensive parasite gene flow not constrained by the political border.^49^^,^^50^ Analysis of parasite migration patterns within and between the two sides of the international border detected unidirectional migration of parasites from Myanmar to China, providing genetic evidence of parasite migration in the border region. Especially for *P. vivax*, a parasite that can travel long distances by infected migrants as silent liver hypnozoites, there is an urgency to identify the sources and sinks of the parasites to enable timely targeted control. The use of polymorphic antigen markers such as *Pvmsp3α* and *3β* has revealed highly diverse *P. vivax* populations in Western Thailand border despite low endemicity, and detected clonal expansion events in Southern Thailand, likely resulting from relaxed control efforts.^51^^–^^53^ Using microsatellite markers, we found drastically divergent *P. vivax* populations in the Eastern and Western Thailand borders, with the central malaria-free zone as a gene flow barrier.^54^ The possibility to distinguish these parasite populations using as few as four microsatellite markers will simplify the tracking of parasite migration, at least among the Thailand borders. We also found that microsatellites could be used to assess the temporal population changes as a means to monitor the progress of malaria control. Although the genetic diversity of *P. vivax* populations over time may remain high, the decreased multiplicity of infection and increased multilocus linkage disequilibrium may reflect a reduction in the parasite population size.^55^ In Eastern GMS, where *P. vivax* populations are less geographically isolated and genetically distinct, whole-genome sequencing (WGS) and the derived SNP barcode may be necessary to distinguish closely related parasite strains and identify the origins of the parasite.^56^^,^^57^ The genomic information from spatially representative parasite populations would identify potential migration patterns using shared identity-by-descent segments,^56^^,^^58^ providing the scientific basis for enhanced monitoring of parasite introduction by migrant populations.

### Zoonotic *Plasmodium knowlesi* malaria.

Since the first cluster of *Plasmodium knowlesi* malaria cases in humans was reported in 2004 in Malaysian Borneo,^59^ reports of *P. knowlesi* incidence have increased strikingly, including in all countries of the GMS—Thailand,^60^^–^^64^ Laos,^65^^,^^66^ Cambodia,^67^ Myanmar,^68^^,^^69^ and Vietnam.^65^^,^^70^^,^^71^ This wide range of *P. knowlesi* in Southeast Asia largely reflects the distribution of the zoonotic hosts (the long-tailed and pig-tailed macaques) and vectors of the Leucosphyrus group of anopheline mosquitoes.^72^^,^^73^ This parasite is probably historically present in the GMS rather than newly emergent. In recent years, we and others have identified an increasing trend of clinical *P. knowlesi* cases in Thailand.^63^^,^^64^ Increased incidences of *P. knowlesi* are likely due to environmental changes such as deforestation, increased forest-related human activities, and potentially peridomestic transmission.^74^ *Plasmodium knowlesi* diagnosis is challenging^75^—it is often misdiagnosed by microscopy due to its resemblance to *P. malariae* and *P. falciparum,* current RDTs are not sufficiently sensitive to detect *P. knowlesi,* and confirmation requires the use of molecular methods.^61^^,^^76^ Its presence as coinfections with other human malaria parasites and in asymptomatic infections also complicates diagnosis and detection, resulting in an underestimate of its real burden.^61^^,^^65^^,^^68^^,^^69^^,^^71^^,^^77^^,^^78^ Since the regional malaria elimination efforts are meant to target all *Plasmodium* species,^79^ it is also time to consider eliminating *P. knowlesi* and other monkey malaria parasites infecting humans (*P. cynomolgi*, *P. inui*, etc.).^80^^–^^82^ The diverse factors associated with the transmission of these zoonotic malaria parasites present a challenge for their elimination, as conventional vector-based control efforts in the domestic environment are ineffective in protecting against sylvatic transmission. Strategies such as repellent and chemoprophylaxis targeting high-risk populations like forest-goers are advocated to accelerate malaria transmission in the GMS.^31^

## MOSQUITO ECOLOGY AND INSECTICIDE RESISTANCE

### Vector ecology.

Malaria vectors in the GMS consist of many *Anopheles* species with varying abundance and importance in malaria transmission among different geographical regions.^83^^,^^84^ Many vector species are in species complexes, including several morphologically similar species and possibly cryptic species. The abundance, diversity, distribution, survivorship, biting behaviors, and vectorial status of different vectors can be influenced by environmental changes, such as deforestation and extensive use of insecticides in both public health and agricultural sectors. As “forest malaria” is a major contributor to residual malaria incidence,^85^^,^^86^ deforestation and landscape changes will have a significant impact on vector ecology and malaria transmission.^87^ Our study conducted in the China–Myanmar border area showed that adult *An. sinensis* and *An. minimus*, the main malaria vectors in this region, had much higher survivorship in deforested than forested areas.^88^ Deforestation also enhanced the survival of *An. minimus* larvae and accelerated larval development.^89^ Our vector surveillance studies conducted in sentinel sites of China, Myanmar, and Thailand have detected major changes in *Anopheles* composition and seasonal dynamics (Table 1). *An. minimus* was the predominant vector in all the surveys.^30^^,^^90^^–^^92^ Consistent with *An. minimus* being a highly adaptive vector, population genetic analysis revealed similar population genetic structure of past and present *An. minimus* populations and substantial gene flow among different geographical populations.^93^ These studies also revealed increased abundance of other vectors such as *An. annularis* and *An. barbirostris* s.l., some of which may support outdoor transmission.^30^^,^^90^^–^^92^ The vector species composition is further complicated by the presence of morphologically identical cryptic species. In Western Thailand, *An. minimus* A and *An. harrisoni* (*An. minimus* C) are two cryptic species often found in the same locations.^94^ Our recent molecular studies of *An. minimus* species collected from Western Thailand showed that ∼11% of the morphologically identified *An. minimus* belonged to a cryptic species (lineage B), which deserves further investigation to understand its bionomics, vectorial status, and species evolution.^93^

**Table 1:** *Anopheles* species compositions in different study sites and study periods at the international borders of the GMS

Species	China–Myanmar border (2012–2014)^a^		Tak, Thailand (2011–2013)^b^		Tak, Thailand (2015)^c^	
	*N*	%	*N*	%	*N*	%
*An. minimus*	13,038	84.6	1,204	40.3	3,725	49.5
*An. maculatus*	530	3.4	640	21.4	999	13.3
*An. culicifacies*	437	2.8	51	1.7	1054	14.0
*An. vagus*	220	1.4	13	0.4	38	0.5
*An. sinensis*	161	1.0	1	–	–	–
*An. barbirostris*	133	0.9	105	3.5	185	2.5
*An. paeditaeniatus*	127	0.8	63	2.1	102	1.4
*An. kochi*	39	0.3	161	5.4	41	0.6
*An. tessellatus*	39	0.3	157	5.3	97	1.3
*An. annularis*	7	0.0	431	14.4	851	11.3
*An. jeypariensis*	277	1.8	–	–	–	–
*An. splendidus*	237	1.5	–	–	–	–
*An. varuna*	–	–	41	1.4	3	0.0
*An. sawadwongporni*	–	–	1	0.0	293	3.9
Other *Anopheles*	175	1.1	118	4.0	133	1.8
Total	15,410	100	2,986	100	7,519	100

Mosquitoes were collected by CDC light traps. This table illustrates major changes of primary vector species in different sentinel sites. *Anopheles* species with ≥ 1% abundance were listed by species names, while the rest was summarized as “Other *Anopheles*.”

^a^ From Wang et al.,^92^^ b^ from Sriwichai et al.,^90^^ c^ from Sumruayphol et al.^92^

In addition to changes in vector species, the malaria vectors in the GMS showed different levels of adaptations to the microecology with dramatic variations among villages. Their different seasonal dynamics underlie their roles in malaria transmission in different seasons.^90^^–^^92^ Residual malaria transmission was traced to farm huts and outdoor agriculture sites, where human biting rates were the highest with *An. minimus*, *An. dirus*, and *An. maculatus* as the primary vectors.^30^ In Western Thailand, *An. minimus* and *An. maculatus* are the main vectors during the two annual malaria transmission peaks while *An. minimus* group is the key primary vector in the dry season,^94^ the Maculatus group is most abundant in the wet season with within-group species-specific variations.^91^ Collectively, this knowledge of the species composition, distribution, bionomics, and dynamics in the international border regions is needed to guide vector control efforts.

### Extent, distribution, and mechanisms of insecticide resistance.

Fast emerging and increasing insecticide resistance of malaria vectors has been implicated as a significant threat to malaria prevention by vector control. Understanding the status, distribution, and mechanisms of insecticide resistance in local malaria vector populations is critical for resistance management and effective malaria control and elimination. We have been monitoring the resistance of malaria vectors to multiple insecticides using the WHO tube test in multiple study sites in China, Thailand, and Myanmar since 2011. The two best-known resistance mechanisms (target site resistance and metabolic detoxification) were investigated in field populations of *Anopheles* mosquitoes. High-level resistance to the four major classes of insecticides (pyrethroids, organochlorines, organophosphates, and carbamates) was observed in *An. sinenesis* populations from Southern and Central China,^95^^–^^97^ and the Eastern Coastal region of China.^98^ Three nonsynonymous knockdown resistance (*kdr*) mutations (L1014F, L1014C, and L1014S) were detected at codon L1014 of the para-type sodium channel gene in *An. sinensis* from China, and these *kdr* mutation alleles exhibited a patchy distribution in frequency from Southern to Central China. Near fixation of *kdr* mutation was detected in populations from Central China but no *kdr* mutations were found in Southwestern China, suggesting that *kdr* alone is insufficient to predict pyrethroid resistance.^99^ The G119S mutation of the *ace-1* gene in *An. sinensis* was moderately frequent in Southern and Central China but fixed in the Eastern Coastal region of China.^96^^–^^98^ Recently, high-level resistance to deltamethrin (mortality rate, 40–80%) was observed in multiple *Anopheles* species, including *An. minimus* s.l. from Thailand in 2018, and the two major vector species complexes (*An. hyrcanus s.l.* and *An. barbirostris s.l*.) from Myanmar in 2019 (unpublished data). However, the *kdr* L1014 mutations or the *ace-1* G119S mutation were not detected in any of the *Anopheles* species analyzed from Thailand and Myanmar, suggesting other mechanisms responsible for pyrethroid and organophosphate resistance (unpublished data). The classification and statistical regression analysis found that metabolic detoxification was the most important resistance mechanism, whereas target site insensitivity of L1014 *kdr* mutation played a less critical role.^96^ We have used transcriptome and WGS to identify transcripts and SNPs associated with insecticide resistance.^100^^,^^101^ These studies highlight the complex network of mechanisms conferring resistance to multiple chemical insecticides in mosquito vectors, and it has important implications for designing and implementing improved vector resistance management strategies.

## ANTIMALARIAL DRUG RESISTANCE

ART-based combination therapies (ACTs) are the frontline treatment of *P. falciparum* and are also recommended as a unified treatment of *P. vivax.* The emergence of *P. falciparum* parasites resistant to ART and partner drugs significantly compromised the efficacies of two ACTs—artesunate-mefloquine (AS-MQ) and dihydroartemisinin-piperaquine (DHA-PPQ).^102^^–^^107^ Clinical ART resistance is manifested as delayed parasite clearance with a parasite clearance half-life of > 5.5 hours, compared with ∼2 hours typically associated with ART-sensitive parasites.^8^^,^^108^^–^^110^ Day-3 blood smear parasite-positivity is also a crude measure of ART resistance, with a 10% cutoff for suspected ART resistance.^111^^,^^112^ In vitro, ART resistance is measured by the ring-stage survival assay (RSA), which measures the survival rate of early ring-stage parasites exposed to a 6-hour pulse of 700 nM of DHA, with an RSA value of ≥ 1% considered as an indication of ART resistance.^113^ Genetically, mutations in the propeller domain of the Kelch-domain protein K13 were identified as the key determinants of ART resistance.^114^ Of the > 200 PfK13 mutations identified in the global parasite populations,^115^^,^^116^ many have been confirmed in clinical efficacy studies^111^^,^^117^^,^^118^ while some have been validated genetically for in vitro ART resistance.^119^^–^^122^

### Monitoring clinical efficacy of ACTs in Western GMS.

We have focused our efforts on monitoring the emergence and spread of ART resistance in Myanmar, given its disproportionate malaria burden in the GMS and its bridging position with South Asia. In Northeastern Myanmar bordering China, the evaluation of DHA-PPQ in 71 patients with uncomplicated falciparum malaria in 2012–2013 demonstrated a 42-day cure rate of 100% and a day-3 parasite-positive rate of 7%.^123^ Similarly, we also found a 28-day cure rate of 100% for artemether-lumefantrine in 41 falciparum patients at the Western border of Myanmar in 2015, although the day-3 positivity rate exceeded 10% in the latter study.^124^ Assessment of 44 culture-adapted clinical isolates for RSA demonstrated increased ring survival rates in parasites with PfK13 mutations.^125^ In addition, day-3 parasite-positive isolates had ∼10 times higher RSA values than day-3 parasite-negative isolates. These studies set the stage for using in vivo efficacy study, in vitro RSA, and molecular surveillance as complementary approaches to monitoring ART resistance.

### Longitudinal in vitro drug susceptibility and molecular markers of resistance.

Our efforts over the past decade to procure clinical isolates from the China–Myanmar border area and establish continuous culture have allowed us to follow the dynamics of in vitro drug susceptibility longitudinally.^126^^–^^129^ From these studies, in vitro sensitivities to 4-aminoquinolines, antifolates, and ARTs deserve some attention. Although chloroquine (CQ) has been withdrawn from treating *P. falciparum* malaria for some time, CQ resistance is consistently high, corresponding with the prevailing occurrence of the Dd2-like *pfcrt* genotype, the primary determinant of CQ resistance.^126^^,^^128^ The use of CQ as the frontline treatment of *P. vivax* malaria may have continually exerted collateral selection pressure on the sympatric *P. falciparum*. Similarly, although the antifolate drugs were withdrawn quite some time ago, parasites exhibited continuous increases in resistance to pyrimethamine, and major mutations in the *pfdhfr* and *pfdhps* genes mediating antifolate resistance remain highly prevalent.^126^^,^^128^ The drug that replaced CQ in this region is PPQ monotherapy,^130^ also the partner drug for the commonly used ACT, DHA-PPQ. Despite previous reports of clinical resistance to PPQ and identification of *pfcrt* mutations, which may be associated with PPQ resistance,^131^^,^^132^ recent studies showed that the efficacy of DHA-PPQ for uncomplicated *P. falciparum* malaria remained high.^123^^,^^133^ Parasites collected over the years were relatively susceptible to PPQ with temporal fluctuations in IC_50_ or IC_90_.^129^ We did not observe parasites with either *plasmepsin 2/3* amplification or new *pfcrt* mutations (H97Y, F145I, M343L, and G353V),^129^ which were described in the DHA-PPQ-resistant populations in Cambodia.^134^^–^^138^

### PfK13-mediated and non-PfK13 ART resistance mechanisms.

**Figure 3.**
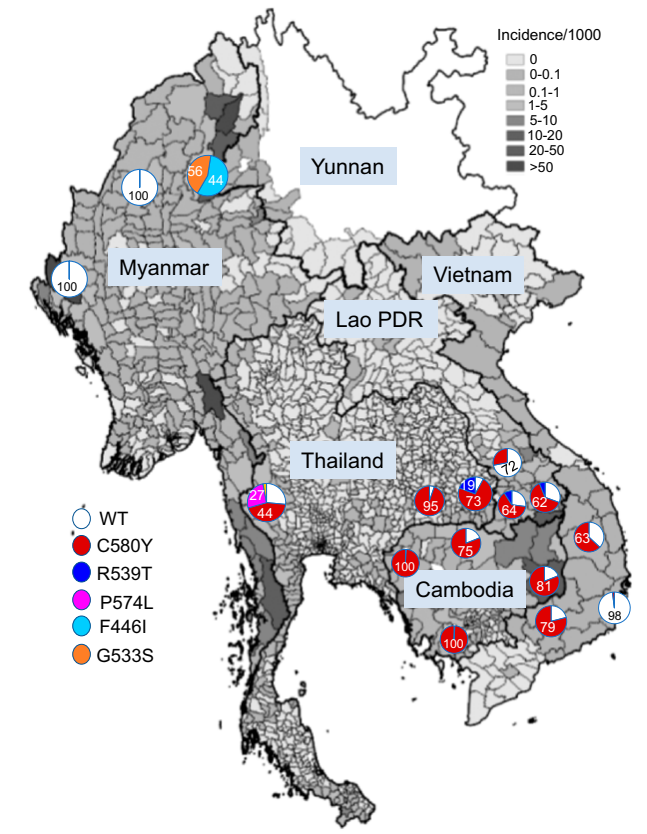
Map of the GMS showing the prevalence and distribution of major PfK13 mutations as pie graphs above the malaria incidence heatmap of the region in 2020. The PfK13 mutation status was updated using malaria parasites collected primarily in 2016 from Cambodia,^122^ Laos,^192^ Vietnam,^193^ Thailand,^194^^,^^195^ and Myanmar.^124^^,^^127^^,^^142^ This figure appears in color at www.ajtmh.org.

PfK13 mutations have also experienced drastic spatiotemporal changes in the GMS. In the Eastern GMS, the C580Y mutation was predominant and has swept rapidly across Cambodia and the Eastern GMS.^114^^,^^115^^,^^122^ In the Western GMS, the F446I mutation is the most prevalent.^139^^–^^141^ Table 2 summarizes the results from our molecular surveillance of PfK13 mutations in the Western GMS. An updated distribution map of major PfK13 mutations in endemic sites of the GMS is shown in Figure 3. On the Eastern border of Myanmar, F446I has gained a steady increase in prevalence between 2007 and 2013.^127^^,^^140^ In the 2014–2016 samples, the G533S mutation emerged and became the second most prevalent at 44%. This new mutation was associated with increased RSA values.^127^ Analysis of asymptomatic *P. falciparum* infections from cross-sectional surveys conducted in the Eastern, Northern, and Western border areas of Myanmar during 2015–2018 detected the F446I mutation only on the Eastern border, suggesting that ART resistance has not spread to or emerged in the Western and Northern borders (Table 2).^142^ To determine whether the PfK13 mutations found in the Western GMS indeed confer ART resistance in vitro, we engineered the F446I, N458Y, C469Y, F495L, and C580Y mutations in the 3D7 background and confirmed that the N458Y and C580Y mutations conferred significant increases in ring survival rate.^121^ Conversely, reverting the F446I, N458Y, C469Y, and C580Y mutations to the wild type (WT) in field isolates resulted in significant decreases in RSA values in all except for the C469Y mutation. Although all tested PfK13 mutations incurred different levels of fitness cost in the transgenic parasites, the F446I and C580Y mutations were almost as fit as the WT,^121^ which may explain their high prevalences in the field parasite populations. In addition, transgenic parasites with these two mutations also exhibited a prolonged ring stage, presumably enabling the parasites to better survive ART treatment, which has a short half-life.

**Table 2:** Amino acid substitutions detected in the *PfK13* gene of *P. falciparum* populations at different border areas of Myanmar

Mutation	China–Myanmar border (and East Myanmar)		Banmauk, North Myanmar	Paletwa, West Myanmar	
	2007–2012 (*N* = 191)^a^	(2013–2016) (*N* = 74)^b^	2017–2018 (*N* = 53)^c^	2015 (*N* = 30)^d^	2017 (*N* = 22)^c^
N11**Y**	1 (0.5)	–	–	–	–
K189**T**	3 (1.6)	2 (2.7)	4 (17.4)	3 (10.0)	–
E252**Q**	1 (0.5)	–	–	–	–
R255**K**	1 (0.5)	–	–	–	–
I352**T**	1 (0.5)	–	–	–	–
I376**V**	2 (1.0)	–	–	–	–
P441**L**	1 (0.5)	–	–	–	–
P443**S**	1 (0.5)	–	–	–	–
F446**I**	52 (27.2)	44 (59.5)	–	–	–
N458**Y**	1 (0.5)	1 (1.4)	–	–	–
C469**Y**	2 (1.0)	–	–	–	–
L492**S**	1 (0.5)	–	–	–	–
F495**L**	2 (1.0)	–	–	–	–
G533**S**	–	15 (20.3)	–	–	–
P574**L**	12 (6.3)	–	–	–	–
C580**Y**	3 (1.6)	–	–	–	–
A676**D**	2 (1.0)	–	–	–	–
H719**N**	2 (1.0)	–	–	–	–
**Total**	88 (46.1)	62 (83.8)	4 (17.4)	3 (10.0)	0 (0)

Letters in bold indicate mutated amino acids. Kelch domain mutations were not found in Northern and Western Myanmar.

^a^ From Wang et al.,^92^^ b^ from Zhang et al.,^127^^ c^ from Zhao et al.,^142^^ d^ from Wu et al.^124^

Investigations into the PfK13-mediated ART resistance mechanisms suggest the involvement of heme-facilitated ART activation and oxidative stress responses.^143^^–^^146^ Since heme is an abundant product of hemoglobin digestion, reduced hemoglobin uptake and digestion would lower ART activation and increase ART resistance. Mutations in the hemoglobinase *falcipain 2a* resulting from in vitro ART selection suggest its involvement in ART resistance.^114^^,^^147^ *Falcipain 2a* harbors geographically divergent mutations, a likely result of drug selection. Analysis of *falcipain 2a* mutation haplotypes in field isolates from the China–Myanmar border area showed that some mutations might reduce the enzyme activity, resulting in increased ART resistance.^148^ Future studies using isogenic parasite lines will better define the role of falcipain 2a in mediating ART resistance.

ART-resistant parasites carrying the WT *PfK13* allele have inspired research on additional players in ART resistance.^149^^,^^150^ The archived clinical isolates have provided the opportunity to perform detailed in vitro studies and uncover the genetic determinants of resistance. Genome-wide association studies (GWAS) of *P. falciparum* isolates collected from the China–Myanmar border area allowed us to identify mutations in genes from multiple pathways such as autophagy (*ATG18*) and DNA repair (*Rad5*) to be associated with increased ART resistance in field isolates.^151^ Further probing into these pathways will determine whether they are directly responsible for ART resistance or constitute background mutations ameliorating fitness costs resulting from causal mutations.^118^

## VIVAX MALARIA TRANSMISSION

As *P. falciparum* incidence declines, eliminating vivax malaria is a major challenge for the “last mile” of malaria elimination in the GMS. The unique biology of *P. vivax—*hypnozoite formation responsible for relapses, early gametocytogenesis enabling transmission before clinical symptoms, and invasion of reticulocytes resulting in low parasitemia—underlies the resilience of this parasite to conventional malaria control measures. In addition, host genetics, drug resistance of the parasite, and changing vector species and populations may also contribute to the persistence and increasing predominance of this parasite.^152^

### Implementation of radical cure.

Currently, PQ is the only drug approved for radical cure of vivax malaria in this region. However, PQ is under-prescribed because it can cause acute hemolytic anemia in patients with glucose-6-dehydrogenase (G6PD) deficiency. In the Kachin ethnicity of Northeast Myanmar, G6PD deficiency reached > 20% prevalence^153^ but patients are not screened routinely for G6PD status before initiating treatment of *P. vivax* malaria. In Thailand and Myanmar, the Mahidol variant (487G>A) is the most predominant and often accounts for ∼90% of all mutations.^153^^–^^156^ The Mahidol variant is associated with different levels of protection against vivax malaria.^157^^,^^158^ Although it is classified as a mild-deficient variant with 30–60% enzyme activity,^159^ some patients with this variant could have < 1% of the normal G6PD activity.^155^^,^^160^^–^^162^ Thus, PQ administration in these patients carries a significant risk of severe hemolysis. We have documented a clinical case of severe hemolysis in a vivax patient after receiving a 3-day low dose PQ (0.25 mg/kg/day) that required blood transfusion.^163^ In Northeast Myanmar, where the G6PD Mahidol variant is prevalent, we conducted an observational study in a cohort of 152 vivax patients to follow the risk of acute hemolysis after treatment with the standard CQ and 14-day PQ regimen. We found that almost 1/3 of the patients experienced clinically concerning declines in hemoglobin, with five requiring blood transfusion (unpublished). Risk in this area is likely exacerbated by preexisting anemia due to host genetics, such as thalassemia and hemoglobin E and other factors, such as helminth infections and poor nutrition.^162^ Thus, the standard 14-day PQ regimen carries a significant risk of acute hemolytic anemia in vivax patients without G6PD testing in Northeast Myanmar.

Another host factor that affects the effectiveness of PQ for radical cure of vivax malaria is the hepatic enzyme cytochrome P450 (CYP) 2D6,^164^ which mediates activation of PQ to its active metabolite(s).^165^^,^^166^ In clinical trials to assess the effectiveness of PQ for preventing relapses, treatment failures were associated with impaired CYP2D6 function.^167^^–^^169^ In Southern China, where malaria has recently been eliminated, malaria importation is a concern, and relapsing malaria has steadily increased in the proportion of the imported cases.^170^^,^^171^ We identified a clinical *P. vivax* case with multiple relapses, potentially due to poor metabolism of the CYP2D6 enzyme.^172^ Thus, the knowledge of the prevalence of low metabolizer CYP2D6 variants in a population is a prerequisite for planning large-scale PQ administration for vivax malaria elimination.

### Chloroquine efficacy and drug resistance.

Chloroquine remains the mainstay treatment of blood-stage *P. vivax* infections in the GMS, though a unified ACT for both *P. falciparum* and *P. vivax* is advocated.^173^ Clinical failures of CQ treatment of vivax malaria are reported sporadically in the GMS,^174^^–^^177^ making efficacy monitoring imperative. Our studies in Northeastern Myanmar also detected declining efficacy of CQ for vivax malaria, with the detection of cases where CQ/PQ treatment failed to clear parasitemia within 7 days, suggesting high-grade resistance.^178^^,^^179^ Given the schizonticidal activity of PQ, these studies may underestimate the CQ resistance status. We also monitored the susceptibilities of the *P. vivax* clinical isolates to a panel of commonly used antimalarials using an ex vivo assay from 2012 to 2016.^180^ For CQ, parasites displayed a wide range of susceptibility, including > 10% parasites with IC_50_ values exceeding 220 nM, a cutoff value used to define CQ resistance.^181^^,^^182^ Only the median IC_50_ values for pyronaridine had an increasing trend from 2.9 in 2012–2013 to 15.5 nM in 2016.^181^^,^^182^ The latter value was much greater than that reported for *P. vivax* parasites from Papua, Indonesia.^183^

To date, the molecular mechanism of *P. vivax* CQ resistance remains unknown. Studies have focused on *pvmdr1* gene, which has geographically divergent nonsynonymous SNPs.^184^ Within the GMS, *pvmdr1* also showed a significant spatial difference in the prevalence of mutations.^185^^–^^188^ The *pvmdr1* Y976F mutation was highly prevalent in Cambodian parasites but was either absent or less frequent in samples from Thailand, the China–Myanmar border region, and Myanmar.^187^^–^^191^ Longitudinal molecular surveillance at the China–Myanmar border showed that the Y976F and F1076L prevalences showed an opposite trend.^186^ The Y976F mutation was present at a moderate frequency of 18.5% in 2008 but sharply decreased to 1.5% in 2012 and completely disappeared in 2015. In contrast, the F1076L mutation continually increased from 33.3% in 2008 to 41.7% in 2012–2013 and 77.8% in 2015. However, we did not detect an association between these two mutations with CQ resistance.^180^^,^^182^ With evidence of the emerging CQ resistance in this region, it is imperative to continuously monitor in vivo and ex vivo CQ sensitivities, coupling these with genetic studies such as GWAS to elucidate the resistance mechanisms.

## CONCLUSION AND FUTURE WORK

With the scale-up of malaria control efforts in the GMS regional malaria elimination campaign, malaria epidemiology has experienced drastic changes with varying degrees of reduction in malaria incidence in the regional countries. Regardless, border regions continue to have persistent malaria transmission, with cross-border introduction constituting a critical threat to malaria elimination. Rigorous surveillance of malaria in border townships needs to be maintained so that real-time information can guide the implementation of existing and new elimination strategies. Vector control measures effectively suppress malaria outbreaks and need to be implemented or strengthened in high-incidence areas. These measures must be regularly adjusted in response to changing prevalence and behaviors of primary vector species and resistance to popular pesticides. There must also be close phenotypic and molecular monitoring of insecticide resistance, especially in areas where insecticide resistance is emerging, such as Western Thailand. Although *P. falciparum* malaria incidence continues to decline, and there is no indication of the emergence or spread of ART-resistant parasites in the Western GMS, continuous studies are still required to identify novel resistance mechanisms, determine ACT efficacy, and monitor the spread of ART resistance. The dominant status of *P. vivax* requires the development and implementation of effective control measures, such as mass drug administration of PQ in combination with mass screening for G6PD deficiency to eliminate the liver stages. New strategies also need to be implemented to determine the burden of zoonotic malaria, understand the ecology of transmission, and identify the high-risk population for targeted prevention. In sum, continued research will help develop integrated tools for countries to move from low or very low endemicity to complete elimination.
